# Patient Preferences for Telemedicine Video Backgrounds

**DOI:** 10.1001/jamanetworkopen.2024.11512

**Published:** 2024-05-15

**Authors:** Nathan Houchens, Sanjay Saint, Latoya Kuhn, David Ratz, Jason M. Engle, Jennifer Meddings

**Affiliations:** 1 Medicine Service, Veterans Affairs Ann Arbor Healthcare System, Ann Arbor, Michigan; 2 Department of Internal Medicine, University of Michigan Medical School, Ann Arbor; 3 Center for Clinical Management Research, Veterans Affairs Ann Arbor Healthcare System, Ann Arbor, Michigan; 4 Department of Pediatrics, University of Michigan Medical School, Ann Arbor

## Abstract

This cross-sectional study assesses patient preferences for various visual backgrounds during telemedicine video visits.

## Introduction

The COVID-19 pandemic prompted rapid adoption of telemedicine. Most physicians had no training on effective webside manner,^1^ including their physical environment. Strategies for optimal visual elements during telemedicine visits have been based on professional expertise and not empirical data.^2,3^ The preferred environment from which a physician conducts video visits remains unknown. Thus, we assessed patient preferences for various visual backgrounds during video visits.

## Methods 

This cross-sectional study was approved by the University of Michigan and Veterans Affairs Ann Arbor Healthcare System institutional review boards. Survey completion implied consent. We followed the STROBE reporting guideline.

Data collection occurred between February 22 and October 21, 2022. Participants included a random sample of adults 18 years or older who had completed an in-person or virtual outpatient visit within the prior year at either institution. Race and ethnicity data were collected but not reported to protect confidentiality (given the majority of participants were White) and because this study was not powered to use these data in the analyses. Additional participants included registrants from a digital health research recruitment platform.

Paper and electronic surveys included photographs of a model physician in different environments (eFigure in Supplement 1). Patients selected their preferred environment, and a composite score was calculated across 6 domains (how knowledgeable, trustworthy, caring, approachable, and professional the physician appeared, and how comfortable the physician made the respondent feel). Scores ranged from 1 to 10, with higher scores indicating greater preference.

Descriptive statistics were used to tabulate results. Mean composite score differences were assessed using linear regression, with a solid color background as the reference category. Differences in preferred environment for all physician types were assessed using multinomial logistic regression. Questions assessed 4 separate physician types (new and established primary care and new and established specialty); these questions were analyzed together, and standard errors were adjusted for repeated measures within participants. Statistical analyses were performed using SAS, version 9.4 (SAS Institute Inc). A 2-sided *P* < .05 was considered significant.

## Results

**Figure 1.**
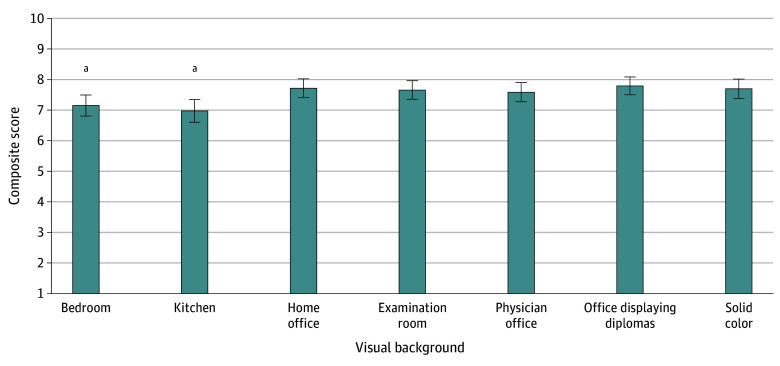
Preferences for Virtual Video Visit Background Environments Higher scores indicate greater patient preference. The reference is the solid color background. Whiskers indicate SDs. ^a^Statistically significant when compared with the reference (*P* < .05).

A total of 1213 patients returned surveys (response rates: university paper survey, 30%; veteran paper survey, 27%; university electronic survey, unknown); 637 patients (54.1%) were 65 years or older; 626 (53.3%) self-identified as female and 544 (46.3%) as male; and 28 (2.4%) self-identified as Asian, 91 (7.9%) as Black, 978 (84.7%) as White, and 57 (4.9%) as multiracial or other (including American Indian, Alaska Native, Arab or Arab American, Native Hawaiian, and other). The solid color background garnered a mean (SD) composite score of 7.7 (2.1). Other professional backgrounds (Figure 1) received similar scores. The physician office displaying diplomas was rated highest across 5 domains (mean [SD] composite score, 7.8 [1.9]). Significantly lower mean (SD) scores were calculated for the bedroom (7.2 [2.3]; *P* = .02) and kitchen (7.0 [2.5]; *P* = .002) environments.

**Figure 2.**
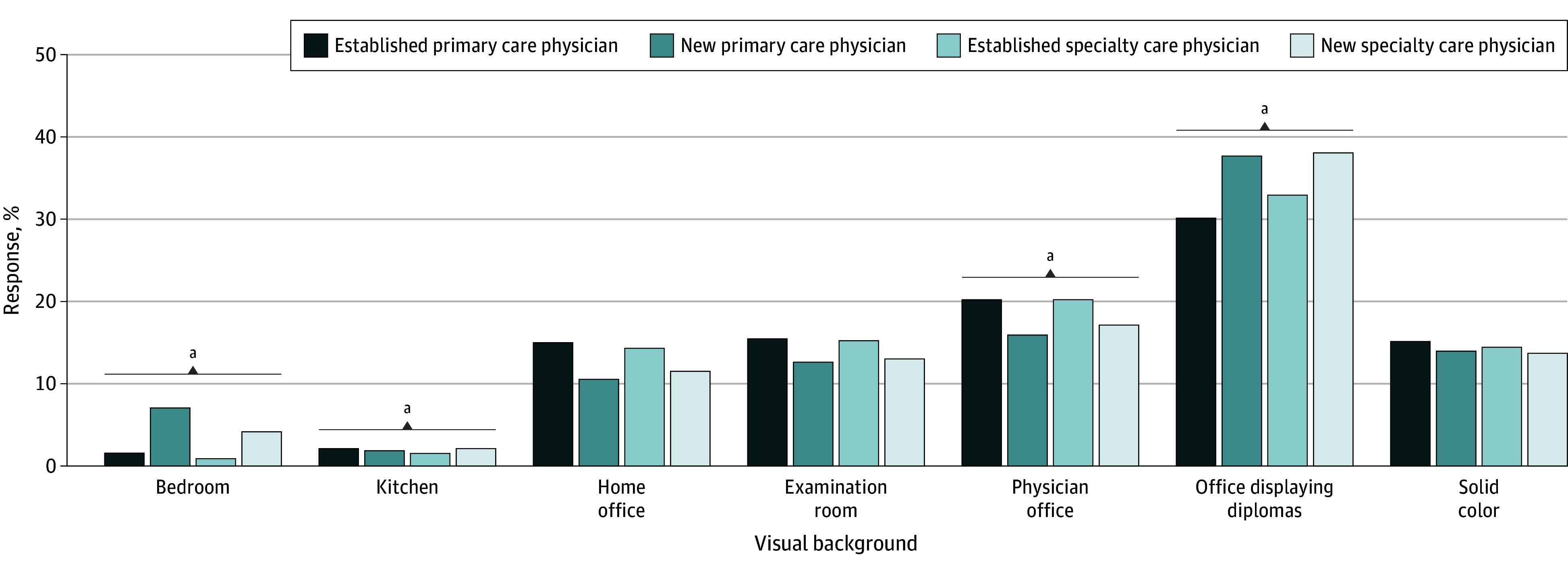
Preferences for Virtual Video Visit Background Environments by Physician Type All physician types in each background are compared with all physician types in the reference category (solid color background). ^a^Statistically significant when compared with the reference (*P* < .05).

The physician office displaying diplomas scored highest for all physician types. Considering all physician types together (a single respondent could choose a different preferred background for different physician types) and comparing with a solid color background (14.4%), respondents significantly preferred physician office (18.4%; *P* = .007) and physician office displaying diplomas (34.7%; *P* < .001) but significantly fewer preferred the bedroom (3.5%; *P* < .001) and kitchen (2.0%; *P* < .001) backgrounds (Figure 2).

## Discussion

In this study, two-thirds of participants preferred a traditional health care setting background for video visits with any physician type, with physician office displaying diplomas rated highest. This background scored similarly to other traditional environments, whereas bedroom and kitchen were significantly less preferred.

Numerous studies have found nonverbal communication to be a modifiable determinant of patient trust and satisfaction.^4,5,6^ To our knowledge, this is the first study to examine patient preferences for the physician’s visual background. Limitations include low response rates for mailed surveys, emphasis on only 1 aspect of telemedicine encounters, and a focus on 2 institutions in 1 geographic region, which may affect generalizability. Nonetheless, findings suggest that patients may harbor specific preferences regarding the background environment used during telemedicine visits. Health care systems should prioritize performing telemedicine visits within a traditional office or examination room environment.

## Notes

Supplement 1. **eFigure.** Photographs of the 7 Environmental Types

Supplement 2.
Data Sharing Statement
