# Supplementary material for: Patient Preferences for Telemedicine Video Backgrounds
Source: JAMA Netw Open. 2024 May 15;7(5):e2411512. doi: 10.1001/jamanetworkopen.2024.11512 (PMC11096986; doi:10.1001/jamanetworkopen.2024.11512)
Supplement: Supplement 1. — eFigure. Photographs of the 7 Environmental Types [file jamanetwopen-e2411512-s001.pdf]

Supplemental Online Content

Houchens N, Saint S, Kuhn L, Ratz D, Engle JM, Meddings J. Patient preferences for telemedicine video backgrounds. *JAMA Netw Open*. 2024;7(5):e2411512. doi:10.1001/jamanetworkopen.2024.11512

eFigure. Photographs of the 7 Environmental Types

This supplemental material has been provided by the authors to give readers additional information about their work.

© 2024 Houchens N et al. *JAMA Network Open.*

eFigure. Photographs of the 7 Environment Types

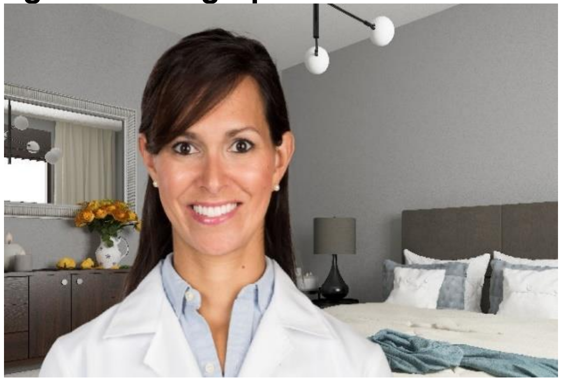

Bedroom

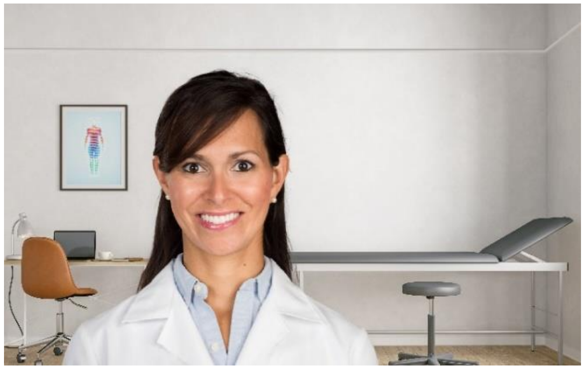

Examination room

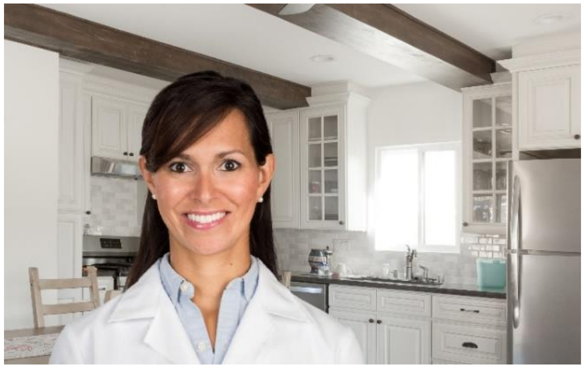

Kitchen

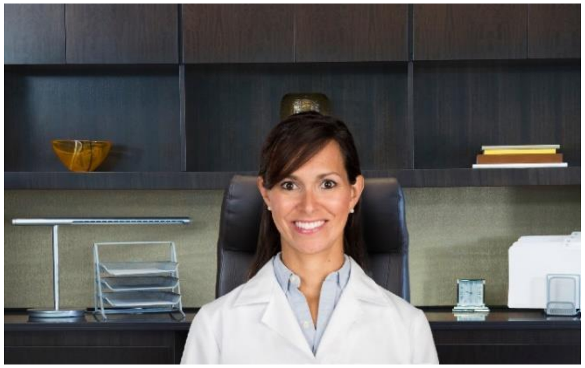

Physician office

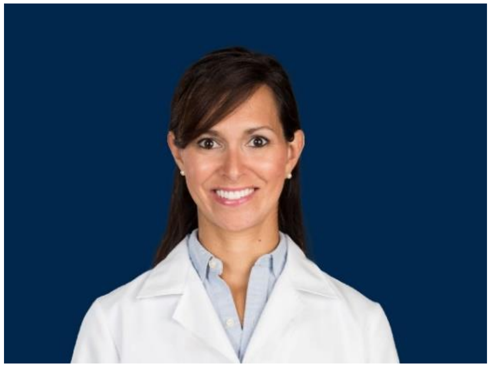

Solid color

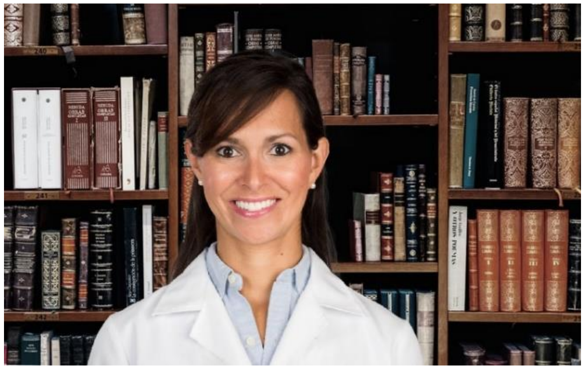

Home office

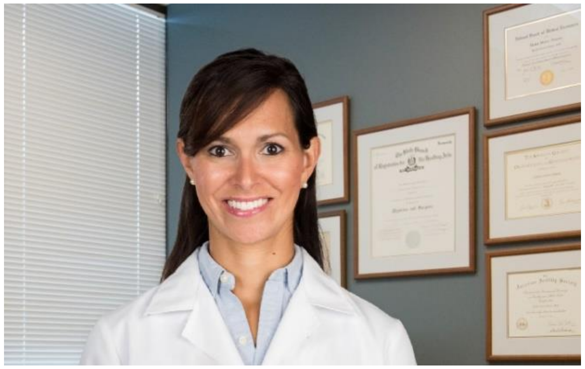

Office displaying diplomas

© 2024 Houchens N et al. *JAMA Network Open.*
