# Supplementary material for: Patient Preferences for Telemedicine Video Backgrounds
Source: JAMA Netw Open. 2024 May 15;7(5):e2411512. doi: 10.1001/jamanetworkopen.2024.11512 (PMC11096986; doi:10.1001/jamanetworkopen.2024.11512)
Supplement: Supplement 2. — Data Sharing Statement [file jamanetwopen-e2411512-s002.pdf]

Data Sharing Statement

Houchens. Patient Preferences for Telemedicine Video Backgrounds. *JAMA Netw Open*. Published May 15, 2024. doi:10.1001/jamanetworkopen.2024.11512

Data
Data available: No
